# Targeting connexins with Gap27 during cold storage of the human donor uterus protects against cell death

**DOI:** 10.1371/journal.pone.0243663

**Published:** 2020-12-10

**Authors:** Katarzyna J. Szymanska, Menekse Göker, Melissa Bol, Jo Van Dorpe, Steven Weyers, Luc Leybaert

**Affiliations:** 1 Department of Basic and Applied Medical Sciences, Ghent University, Ghent, Belgium; 2 Department of Obstetrics and Gynecology, Ghent University, Ghent, Belgium; 3 Department of Pathology, Ghent University, Ghent, Belgium; University of Cambridge, UNITED KINGDOM

## Abstract

Uterus transplantation is an experimental infertility treatment for women with uterine factor infertility. During donor uterus retrieval and subsequent storage, ischemia and other stressors are likely to occur, resulting in the delayed restoration of organ function and increased graft rejection. The uterus expresses connexin-based hemichannels, the opening of which can promote ischemic cell death, as well as gap junctions that may expand cell death by bystander signaling. We investigated if connexin channel inhibition with connexin channel inhibitor Gap27 could protect the uterus against cell death during the storage period. The study involved 9 female patients undergoing gender-change surgery. Before uterus removal, it was exposed to *in situ* warm ischemia with or without reperfusion. Uterus biopsies were taken before, during, and after ischemia, with or without reperfusion, and were subsequently stored under cold (4ᵒC) or warm (37ᵒC) conditions. TUNEL cell death assay was done at various time points along the combined *in vivo*/*ex vivo* experimental timeline. We found that Gap27 protected against storage-related cell death under cold but not warm conditions when the uterus had experienced *in situ* ischemia/reperfusion. For *in situ* brief ischemia without reperfusion, Gap27 reduction of cell death was delayed and significantly less, suggesting that protection critically depends on processes initiated when the organ was still in the donor. Thus, the inclusion of the connexin channel inhibitor Gap27 during cold storage protects the uterus against cell death, and the degree of protection depends on the history of exposure to warm ischemia. Gap27 protection may be indicated for uteri from deceased donors, in which ischemia is likely because life-saving organs have retrieval priority.

## Introduction

Uterus transplantation is an experimental infertility treatment option for women with a dysfunctional or absent uterus, also known as absolute uterine factor infertility. After extensive research on several animal species [1], Brännström’s team described the first human delivery after uterus transplantation from a living donor in 2014, and since then, more live births were recorded [2–6]. The retrieval of the uterus from a living donor has the advantage that the organ can be examined for uterine and cervical pathologies before transplantation, however, the donor patient faces surgical and postoperative risks. Deceased and cadaveric donors may bring additional difficulties because the timing of the procedure is dependent on the availability of the suitable donor, and on the organ retrieval priority from a multi-organ donor. The first human uterus transplantation from a multi-organ brain-dead donor was performed by Özkan, where the uterus was the first organ to be retrieved, creating the best conditions to prevent cell injury related to warm ischemia [7]. The first live births after uterus transplantation from multi-organ deceased donors were described recently in Brazil and in the US [6, 8]. The success of uterus transplantation may be further increased by applying treatments that minimize ischemia-reperfusion (further abbreviated to I/R) injury [1]. Long ischemic exposures and reperfusion injury are indeed associated with impaired post-transplantation perfusion, delayed graft function, and increased frequency of acute or chronic rejection. During the transplantation process, both cold ischemia (in the period between flushing and the start of anastomosis surgery), as well as warm ischemia (during anastomosis surgery), can occur [9], which may lead to cell injury followed by apoptotic or necrotic cell death. Once perfusion is re-established, reperfusion injury may aggravate things [10]. Ischemia, as well as I/R injury, are two conditions well known to involve connexin-linked injury mechanisms, which include the expansion of cell death via gap junction channels and the opening of hemichannels that by themselves may lead to cell injury/death [11–14]. Connexins are membrane proteins that form gap junctions and hemichannels and play a key role in the development and function of the female reproductive system (reviewed in [15]). Gap junctions enable the direct and selective exchange of chemical/metabolic signals between cells [16, 17] but under pathological conditions such as I/R, gap junctions may expand cell death by spreading cell death signals between neighboring cells [12]. On the other hand, gap junctions may act protectively as well by distributing and supplying cells with nutrients, metabolites, and survival messengers [18]. Hemichannels open during cellular stress conditions [11, 19] creating a non-selective leakage pore, through which atomic ions and essential metabolites can escape from the cell leading to cell damage and cell death [18, 20]. Several studies have demonstrated that blocking connexin channels, in particular hemichannels may limit I/R injury in isolated cardiomyocytes as well as the intact heart [21, 22]. Moreover, we previously showed that Gap27, a short sequence identical to the second extracellular loop of Cx43 protects human blood vessels against cryopreservation-induced cell death in smooth muscle cells [23].

Here, we investigated whether connexin channel inhibition with Gap27 during the storage period between donor uterus retrieval and its implantation in the recipient patient, could improve the uterus condition in terms of cell death scoring. We tested both cold (4°C) and warm (37°C) static storage conditions and also checked whether warm I/R applied to the uterus *in situ* while still in the donor, influenced the uterus outcome after storage.

## Materials and methods

### Patients

Tissue samples were taken from the uteri of healthy transgender patients of Western European descent undergoing a laparoscopic hysterectomy and ovariectomy. Transgender patients in the age range of 18–27 years were included. Patients with complex medical history like diabetes, heart and lung disease or thrombo-embolic disease were excluded from the study; no organs were procured from prisoners. In total, 9 uterus donors were recruited in the study, 4 in Experiment 1 (recruitment from December 2014 to January 2016) and 5 in Experiment 2 (recruitment from July 2017 until April 2018). The donors were females, in transition; their mean age was 21.6 years old (median age 22), 7 were still studying and two were working, one patient was from Holland, and eight from Belgium. The recruitment took place during outpatient clinic visit, transgender patients planning a surgery were asked to participate in this study after a thorough explanation. The recruitment was done in the Ghent University Hospital and the research was done in the Department of Basic and Applied Medical Sciences, Ghent University.

Before the surgery, the patients were hormonally treated for approximately a year with Nebido^®^.

### Ethical approval

All material from patients was collected after their written informed consent before surgery according to a protocol that was approved by the ethical committee of the Ghent University Hospital. All the experiments were carried out in accordance with the protocol approved by the ethical committee of the Ghent University Hospital.

### Experiment 1

#### *In situ* warm I/R and uterus biopsy sampling

**Fig 1:**
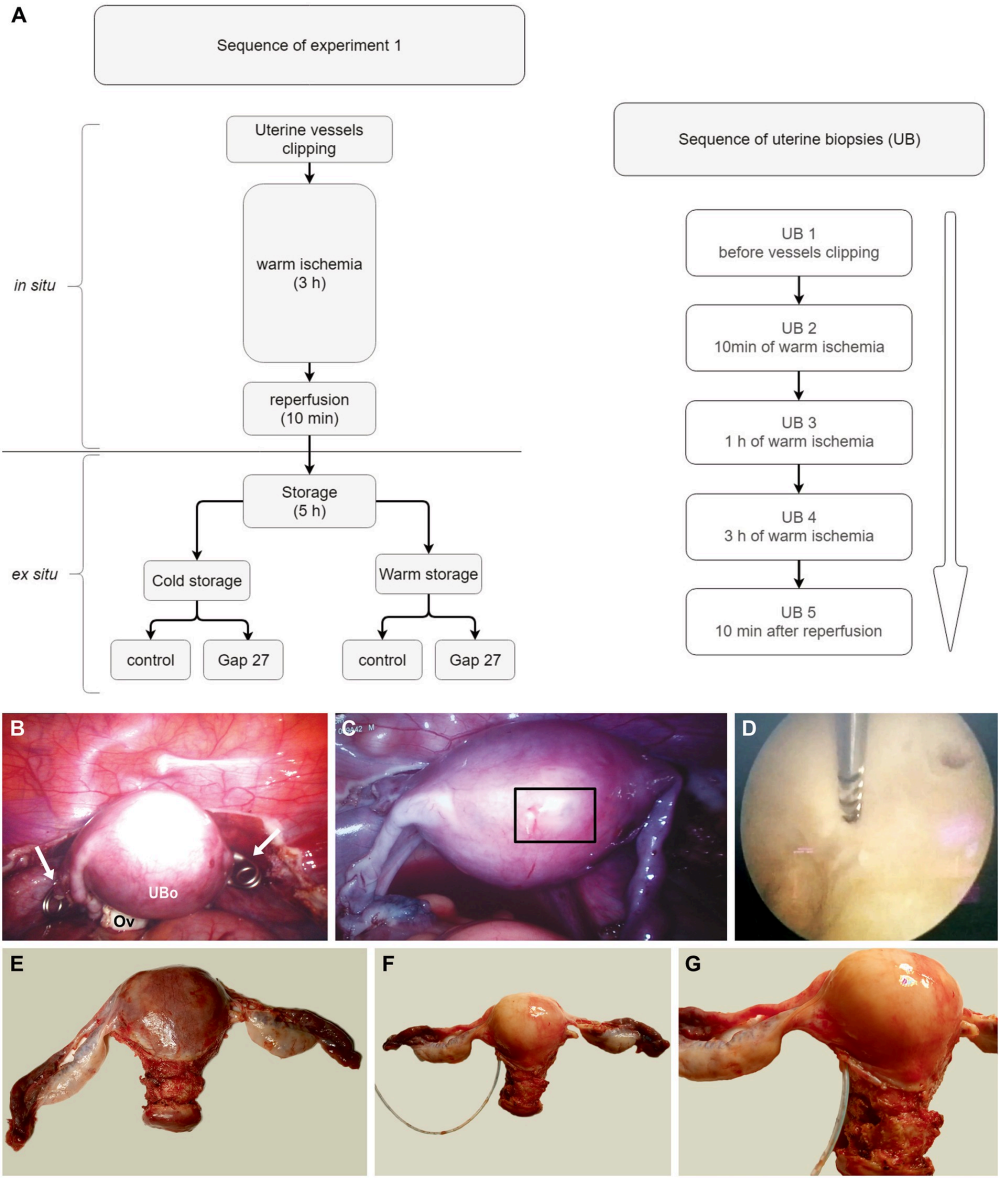
Design of Experiment 1. **A**. The protocol of Experiment 1 (left) and the sequence of the uterine biopsies (right). **B-G** Pictures illustrating the uterus during warm ischemia and after hysterectomy. (B) Clipping of the uterine arteries and veins using two surgical clips (black arrows). UBo, uterine body; Ov, ovary. (C) Uterus during the first (UB1) and second biopsy (UB2) taken at the site indicated by the rectangle. (D) Intrauterine image during biopsy of UB2. (E) Uterus before flushing with Custodiol^®^. (F) Uterus after flushing before taking samples for cold/warm storage. (G) Uterus after flushing, depicting the catheter entry site.

Uterus biopsies were taken in transgender patients (N = 4) during laparoscopic hysterectomy and ovariectomy combined with mastectomy. The hysterectomy was performed to near completion except for the ligation and transection of the uterine arteries and veins. Each of the four patients had five uterine biopsies taken during the surgery; the first uterine biopsy (UB1), was then taken before bilateral clipping of the uterine arteries and veins, the second (UB2), 10 min after clipping, corresponding to 10 min of warm ischemia. The third biopsy (UB3) was taken after 1 h of warm ischemia and the fourth (UB4) just before removing the vessel clips, corresponding to 3 h of warm ischemia. During these 3 h of *in situ* warm ischemia, plastic surgeons performed the bilateral mastectomy. Upon finishing the mastectomy, the uterine vessel clips were removed. The last biopsy (UB5) was taken 10 min after reperfusion; thus, warm I/R involved 3 h of ischemia followed by 10 min of reperfusion. All biopsies during surgery were hysteroscopically guided, taken with a Spirotome Cervicore device (Cervical Macro Biopsy System, 14 G x 350 nm, Medinvents, Belgium) and had approximately 1.6 mm in diameter. The uterine biopsies were taken from the fundus of the uterus, along an inside to outside direction (endometrium to myometrium) over the thickness of the wall. Fig 1B–1D shows the clipping of the uterus and the procedure of taking biopsies.

#### *Ex-situ* biopsy sampling and storage

After the uterus was removed from the patient (N = 3, samples from one patient were not processed according to our storage protocol due to peptide solubility problem), it was flushed three times (500 ml) with cooled Custodiol^®^ perfusion solution (Histidine-Tryptophan-Ketoglutarate, HTK solution, Franz Köhler Chemie GmbH, Germany) to wash the organ from the blood (see Fig 1E–1G). Custodiol^®^ is often used for heart cold storage, which, like the uterus, consists mainly of muscle cells. Storage in Custodiol^®^ was suggested to be superior to other solutions [9, 24, 25] but there are currently no hard data to support such claim. Here, we chose Custodiol^®^ because of its musculoplegic properties.

Uterus biopsies (3 biopsies per patient per storage condition) were collected immediately after that (see Fig 1A *ex situ*) with a Biopsy Punch (6 mm in diameter, Stiefel, Belgium). *Ex-situ* biopsies were kept in small containers filled up with Custodiol^®^ solution with or without Gap27 peptide either in the fridge (4°C) or warm incubator (37°C), to mimic cold or warm storage conditions. After storage, samples were prepared as described under *Sample preparation and TUNEL staining*.

### Experiment 2

#### *In-situ* short warm ischemia, biopsy sampling and cold storage

**Fig 2:**
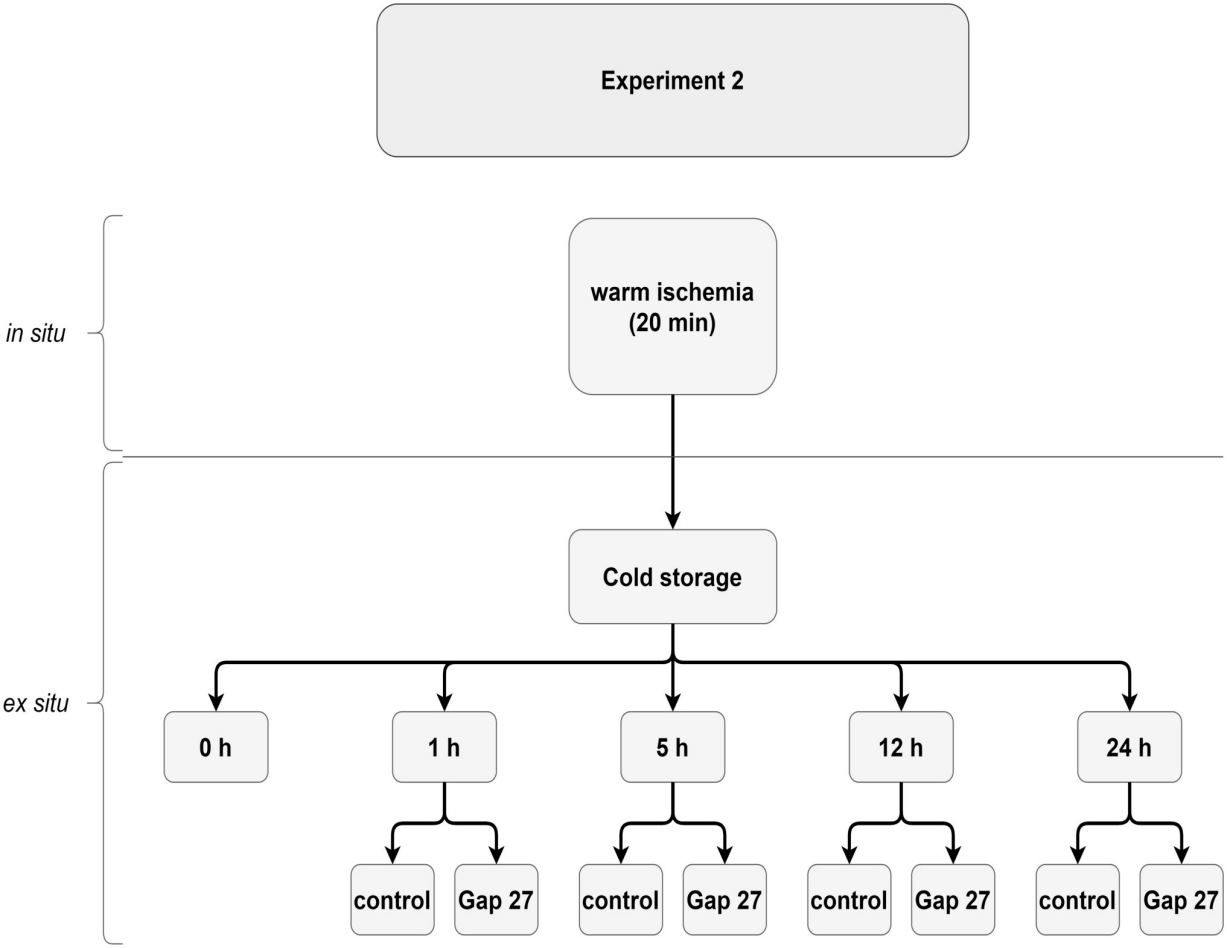
Design of Experiment 2. The uterine arteries were clipped *in situ* for 20 min prior organ retrieval. After the uterus was removed, uterine samples were collected and stored up to 24 h in cold storage conditions (4°C) with or without Gap27 peptide.

We investigated cell death during cold storage of uterus biopsies after *in situ* exposure to 20 min warm ischemia (Fig 2) induced by clipping the uterine arteries as was done in Experiment 1. The uterus was then removed from the patients (N = 5), without reperfusion and was flushed *ex-situ* three times with Custodiol^®^ perfusion solution as was done for the warm I/R condition of Experiment 1. Uterus biopsies (n = 5, one biopsy from 1 patient per storage condition) were only collected after organ retrieval with a Biopsy Punch (6 mm diameter, Stiefel, Belgium) and were kept in perfusion solution with or without Gap27 peptide and cold-stored at 4°C for 0 h, 1 h, 5 h, 12 h or 24 h. After storage, biopsy samples were prepared as described under *Sample preparation and TUNEL staining*.

### Sample preparation and TUNEL staining

At the end of the storage period, biopsies were fixed in 4% formaldehyde (VWR, Belgium) at 4°C overnight. After washout, samples were embedded in paraffin and cut into 5 μm sections. On the day of the staining, slides with tissue samples were deparaffinized and permeabilized for 8 min in 0.1% TritonX-100 (VWR, Belgium) dissolved in PBS (Sigma-Aldrich, Belgium). Apoptosis was detected by *in situ* terminal deoxynucleotidyl transferase (TdT)-mediated deoxyuridine triphosphate (dUTP) nick end-labeling (TUNEL), using In Situ Cell Death Detection Kit (Roche, Belgium). Samples were incubated with the TUNEL reaction mixture for 1 h at 37ᵒC in the dark. Samples treated with DNase I (1000 u/ml in H_2_O, Sigma-Aldrich, Belgium) for 10 min were used as a positive control and not exposed to TdT enzyme, as a negative control. Next, samples were washed with PBS and stained with 1 μg/ml DAPI (Life Technologies Europe) in PBS and were mounted with Vectashield mounting medium (Labconsult, Brussels, Belgium). Three random slides per replicate and three images across the sample were acquired with a Nikon TE300 epifluorescence microscope equipped with a 10× objective (Plan APO, NA 0.45; Nikon), and a Nikon DS-Ri1 camera (Nikon BeLux, Zaventem, Belgium). The number of TUNEL-positive cells was expressed relative to the total number of nuclei stained with DAPI. The calculations were performed using FIJI ImageJ after the application of a threshold corresponding to the upper level of the background signal.

### Gap27 peptide treatment

Gap27, a short peptide identical to a sequence on the second extracellular loop of Cx43 (SRPTEKTIFII), was used in this study to block connexin gap junctions and hemichannels during storage of uterus biopsies. Inhibition of gap junctions by Gap27 is characterized by an IC_50_ of 20–30 μM [26] and we used 200 μM to obtain an estimated 87–91% inhibition. The IC_50_ for hemichannel inhibition is ~161 μM (Hill coefficient of 2; [21] giving ~61% inhibition at 200 μM concentration. Previous work demonstrated that 200 μM of Gap27 is indeed sufficient to significantly reduce cell death in human blood vessels after cryopreservation and thawing [23]. Gap27 was synthesized by Pepnome Limited (Jida Zhuhai, China) at >90% purity. The peptide was dissolved in the transplantation solution at a final concentration of 200 μM.

### Data and statistical analysis

Data are expressed as mean ± SEM, with ‘n’ denoting the number of replicates; ‘N’ indicates the number of patients. The number of patients from which samples were taken is specified per experiment. Statistical analysis was performed using GraphPad Prism 7 software (GraphPad Software, San Diego, USA). In Experiment 1, we used one-way ANOVA with Sidak’s multiple comparisons. For Experiment 2, we used a two-way ANOVA with Sidak’s multiple comparisons. The level of statistical significance level was set at p < 0.05.

## Results

### *In situ* warm ischemia/reperfusion and effect of Gap27 on subsequent cold or warm storage conditions

**Fig 3:**
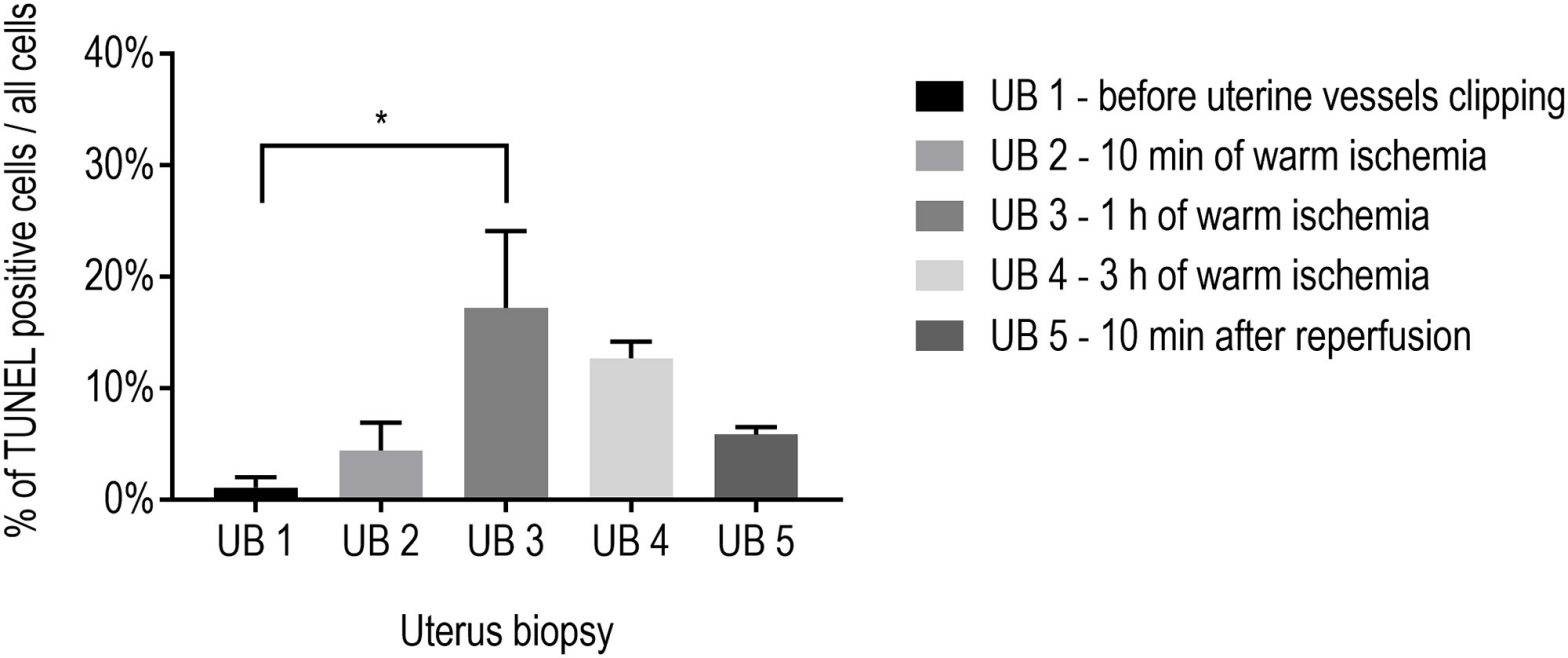
Evolution of TUNEL scoring in uterus biopsies before, during, and after uterine vessel clipping. The imposed warm ischemia significantly increased the number of TUNEL-positive cells, reaching a peak after 1 h of ischemia. After 3 h of ischemia, values decreased and were not different from control before clipping. Following 10 min of reperfusion, TUNEL scores were in the range of the UB2 values. All data are presented as mean ± SEM, * p < 0.05, one-way ANOVA with Sidak’s multiple comparisons, n = 4 from 4 patients.

The study design was divided into two parts: an *in situ* and *ex situ* part, an overview of which is presented in Fig 1A. In *in situ* part of Experiment 1, we investigated cell death as estimated from TUNEL scores in uterus biopsies taken at various time points during 3 h of *in situ* warm ischemia (clipping of uterine arteries and veins in the patient) followed by 10 min of reperfusion (release of clips) (Fig 1A and 1B). Based on the results of four patients (N = 4) with one biopsy sample taken at each time point per patient (n = 4; UB1—UB5), we observed that the highest percentage of TUNEL-positivity was in uterus biopsy taken after 1 h of warm ischemia (UB3), which was significantly above the control value before induction of warm ischemia (p = 0.04; Fig 3). Surprisingly, 3 h of warm ischemia did not trigger significant TUNEL positivity (UB4), nor did 10 min of reperfusion (Fig 3). As a result, the significant TUNEL positivity observed for the UB3 sample appeared transient as the values tended to decrease gradually, and none of those were significantly above control. The disappearance of TUNEL positivity indicates that the cellular energy potential is still able to support DNA repair; such recovery from cell death initiation has been called anastasis [27]. Other publications of reversal of cell death events after DNA damage were documented before [28, 29]. Overall, our findings suggests a rather low sensitivity of the uterus to warm ischemia and absence of reperfusion injury after 10 min of restored circulation.

**Fig 4:**
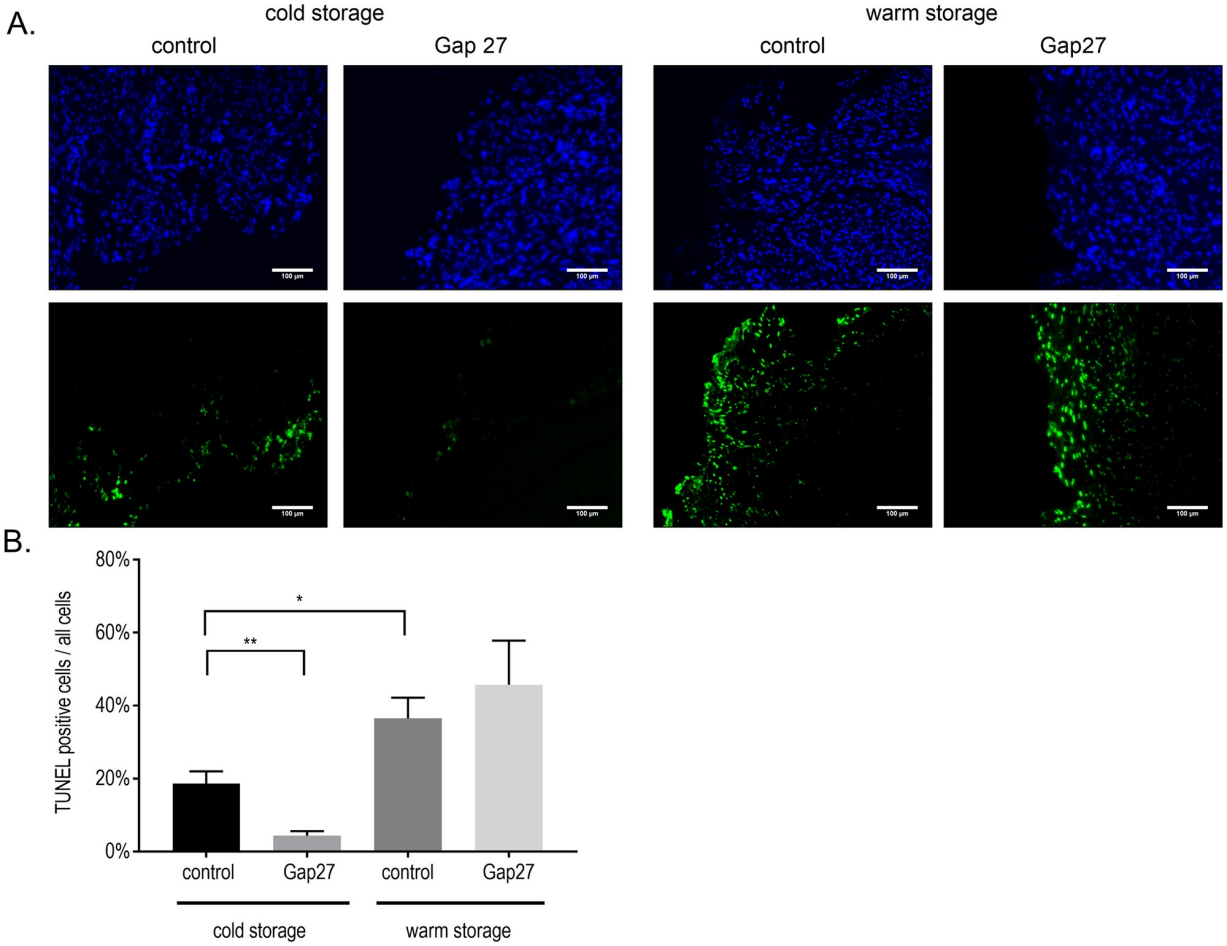
Warm *vs*. cold storage after long warm I/R and the effect of Gap27. A. Representative TUNEL staining images of uterus tissue in the different experimental groups. TUNEL-positivity in green and nuclear DAPI staining in blue. **B**. TUNEL scores after warm storage (37 °C, 5 h) were significantly higher compared to cold storage. The inclusion of Gap27 in the cold storage medium significantly decreased the TUNEL scores. In the warm storage group, Gap27 had no protective effect. * p < 0.05, ** p < 0.005, one-way ANOVA with Sidak’s multiple comparisons of selected bars, n = 9 from 3 patients.

We next tested cell death after 5 h of cold or warm storage. To that purpose, after 3 h of *in situ* warm ischemia and 10 min of reperfusion, the uterus was removed, biopsies were taken and subsequently kept under either cold (4°C) or warm (37°C) storage conditions in Custodiol^®^ solution. We used 3 biopsy samples from each of the 3 donor patients included in this study per storage condition. As observed in Fig 4, TUNEL positive counts after cold storage (18.6% ± 1.9%) were significantly lower (p = 0.03) of those associated with warm storage (36.5% ± 10.7%). Additionally, the inclusion of Gap27 (200 μM) in the cold storage solution, strongly and significantly decreased TUNEL positivity by a factor of 4 (from 18.6 ± 1.9% to 4.35 ± 1.1%; p = 0.004), attaining a value that was in the same order of TUNEL positivity just before the start of the storage procedure (5.5 ± 2.7%, sample UB5, Fig 3). In contrast, Gap27 had no protective effect at all when applied during conditions of warm storage (Fig 4A and 4B).

### *In situ* warm ischemia and effect of Gap27 on subsequent cold storage

**Fig 5:**
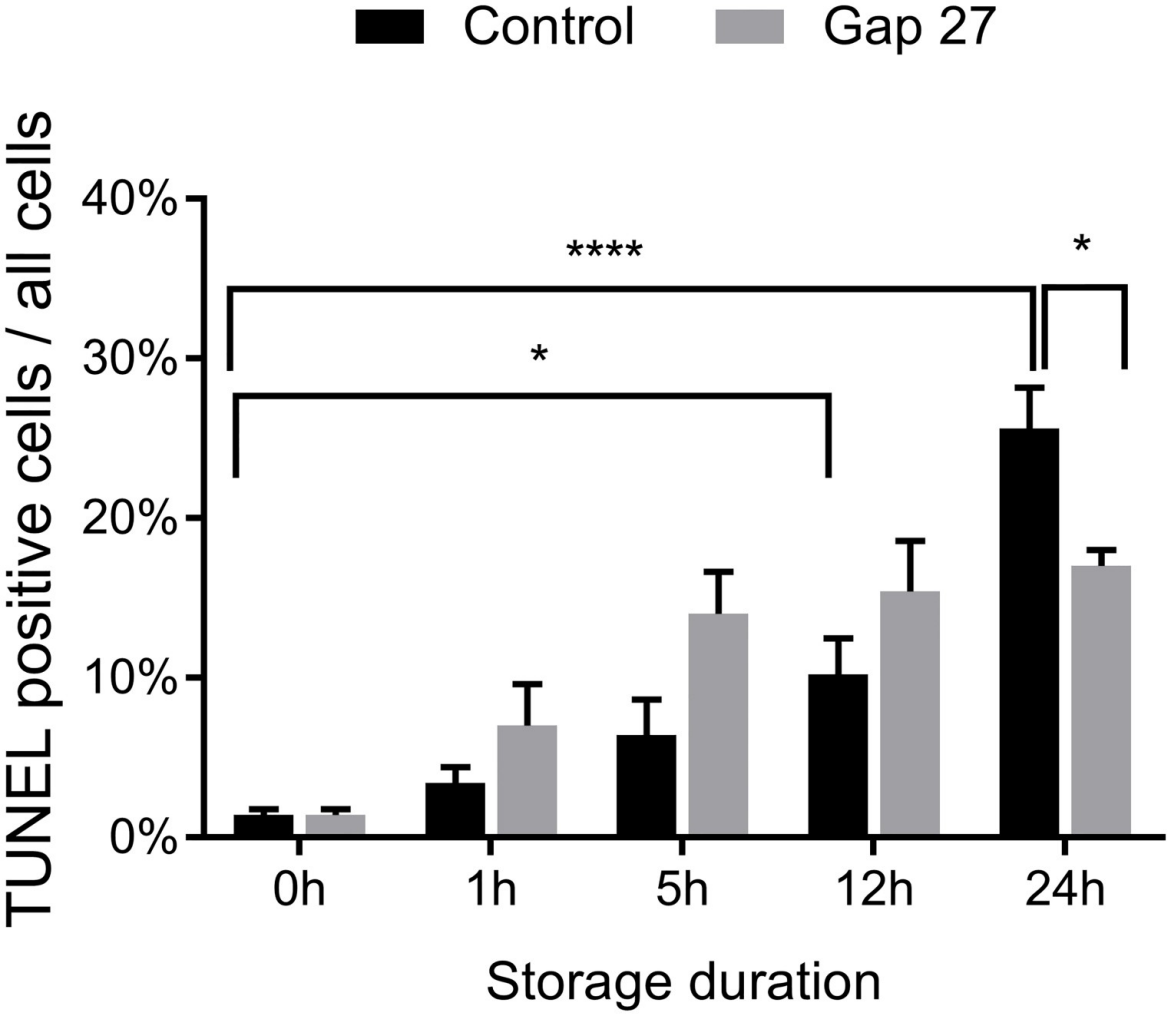
Cold storage after short warm ischemia without reperfusion. Evolution of TUNEL scores during cold storage. After 12 h and 24 h of cold storage, TUNEL scores were significantly increased. Inclusion of Gap27 during cold storage significantly decreased the TUNEL score only for the 24 h storage period. *, # p < 0.05, **** p < 0.0001, two-way ANOVA to assess the effect of time and Gap27, * is the comparison of time points to 0 h and # shows the comparison of Gap27 treatment to control, n = 5 from 5 patients.

Due to a change in the surgical procedure whereupon the mastectomy was not combined anymore with a hysterectomy but done as a separate surgical intervention, application of a long 3 h warm ischemia period was not possible for follow up experiments. Therefore, we adapted the protocol of Experiment 2, and the *in situ* warm ischemia period was reduced to 20 min. Given the fact that reperfusion did not provoke significant cell death in Experiment 1, we further decided not to apply a reperfusion phase for Experiment 2. Because of the more limited time available during surgery, we only took uterus biopsies after removal of the uterus from the body, and its flushing with Custodiol^®^ solution. We then exposed the samples to various periods of cold storage (4°C) up to 24 h, in control, or with Gap27 added to the solution (flowchart of Experiment 2 see Fig 2). We included 5 patients and used 1 biopsy per patient per condition (n = 5). The results demonstrated a gradual increase in TUNEL positivity during cold storage, with only the 12 h and 24 h storage periods attaining statistical significance as compared to the 0 h measurement (p = 0.013 and p < 0.0001 for 12 h and 24 h respectively; Fig 5). The TUNEL score at 5 h cold storage was significantly lower compared to 5 h cold storage after the 3 h of warm I/R protocol of Experiment 1 (first bar of Fig 4B) (8.6 ± 1.5% n = 7 *vs*. 18.6 ± 3.4%; one-tailed t-test p < 0.05), reflecting the shorter *in situ* warm ischemia period (20 min *vs*. 3 h) and the absence of *in situ* reperfusion. Gap27 did not protect as inferred from the TUNEL scores in the first 12 h period, but it significantly reduced TUNEL positivity after 24 h of storage by ~35% (control: 25.4 ± 2.4% *vs*. Gap27: 17.2 ± 1.0%; p = 0.028) (Fig 5).

## Discussion

We evaluated how initial warm ischemia, and subsequent different storage conditions, influenced the viability of the human uterus. We found that Gap27 protects the uterus against cell death, measured by TUNEL staining, during cold storage while it was ineffective against warm storage. Interestingly, protection by Gap27 was stronger and quicker when the uterus had previously experienced warm I/R (3 h/10 min) compared to 20 min ischemia without reperfusion, in which case protection was only detectable after 24 h cold storage.

During ischemia, due to restricted or reduced blood flow, the tissue is deprived of oxygen and blood nutrients, causing a gradual loss of cellular homeodynamics. At reperfusion, these changes induce inflammation and oxidative stress that can lead to an organ’s loss of function [30]. The detrimental effects of warm I/R are well known in solid organ transplantation, and it is crucial to limit ischemia exposure time [31, 32]. Warm ischemia during transplantation usually lasts less than 1 h, but in complicated cases, could rise to 2–3 h.

We observed low sensitivity of the uterus to 3 h *in situ* warm ischemia and the apparent absence of reperfusion injury following restored circulation in the patient. Adachi *et al*., 2016 and Kisu *et al*., 2017 used a similar experimental set-up in non-human primates to investigate the allowable exposure time to warm ischemia induced by clamping the uterine vessels for up to 8 h followed by 3 h of reperfusion [33, 34]. In their studies, light and electron microscopy investigations demonstrated no abnormalities at the organelle level, and menstruation was restored with warm ischemia times of less than 4 h [33, 34]. Brännström et al. reported warm ischemia times in humans are tolerable for at least 1 h 30 min [35, 36]. Our observations correspond with the non-human primate studies and demonstrate that the tolerable ischemic time of human uterus is in the order of 3 h.

Moreover, the *in situ* warm I/R experiment mimics what the uterus would experience during other types of procedures where the uterine artery is temporarily blocked. For example, transient blocking of the uterine perfusion by clipping uterine arteries is used to prevent excessive blood loss during laparoscopic myomectomy [37]. Studies by Wang et at. 2008 and Liu et al. 2011 confirm that blocking uterine perfusion for 1.5–2 h does not affect uterus function [38, 39]. However, in myomectomy surgery, only the uterine artery is clipped while here in our study, we transected all other accessory uterine vessels before clipping the uterine artery and vein.

Apoptotic cell death progressed faster after warm than after cold storage. Moreover, cold storage injury was two-fold higher when the organ was previously exposed to the long *in situ* warm I/R compared to brief warm ischemia. This indicates that warm ischemia initially triggered a cellular stress response which evolves to cell death during subsequent storage. When the organ was retrieved within 20 min, cell death significantly increased only after 12 h of cold storage, which confirms the observations made by Wranning et al. who found an allowable cold storage time of at least 6 h for human uterine smooth muscle tissues in protective solution [9]. Some studies in humans claim that the uterus may be resistant to cold storage for up to 12 h without histological changes [40] or even 24 h based on electron microscopic evaluation [41]. Recently, a case study of the first baby born from a multi-organ deceased donor was published. The total ischemia time was 7 h 50 min, with 6 h 20 min of cold and 1 h 30 min of warm ischemia [8]. However, the study did not specify the cold storage time. Hence, the allowable ischemia times require further scrutiny. Altogether, our results confirm that cold storage is superior to warm storage, and the uterus can withstand cold storage for a longer time.

It has been confirmed that gap junctions play a role in the pathogenesis of ischemia, cold storage, and reperfusion injury in mice [42, 43]. The insult caused by oxidative stress in the regions of disturbed blood flow might be propagated by gap junction channels as cell death signaling molecules spread to neighboring cells by the bystander effect, causing cells or tissue damage amplification and deterioration [44]. Apart from gap junctions, the opening of unpaired hemichannels may also be involved in cell injury (reviewed in [45]), as exemplified by the protective effects of Gap26 and Gap27 in reducing ischemic cell injury in the brain and myocardial I/R in the intact heart [22, 46, 47].

Here, Gap27 strongly protects the uterus against cell death during cold storage when surgery involved pre-exposure to long warm I/R. Thus, Gap27 protection seems to depend on the preceding *in situ* warm ischemia, indicating that the peptide protects against cell death processes that were initiated when the uterus was still in the patient rather than by storage-induced processes. Cell death processes initiated during long, *in situ* warm ischemia, continue during the warm storage. In this case, cell death probably overwhelms the protective potential of Gap27, possibly because other, non-connexin signaling pathways are activated. Collectively, this indicates that the peptide does not primarily target intrinsic storage-linked processes but rather events initiated in the preceding period of uterus retrieval from the donor.

There are several limitations to our study. First, the experimental warm ischemia only represents the first phase during organ retrieval but lacks a second warm ischemia phase that would occur during re-anastomosis upon organ implantation. Second, we did not evaluate the endometrium separately since before surgery, patients went through long hormonal treatment and the endometrial layer was thin and non-active. Third, the number of available patients was low and statistical power suboptimal, making it possible that the results obtained do not necessarily apply to a larger patient cohort, therefore, the results should be interpreted with caution. Lastly, we could not distinguish between apoptotic *vs*. necrotic cell death because the readout was based exclusively on TUNEL staining. Caspase-3 staining was tested, but statistical variability was large, precluding the extraction of accurate data. Further studies evaluating additional readouts are necessary, including uterus morphology, oxidative stress markers and cellular, biochemical or proteomic markers of distinct cell death modes such as necrosis, apoptosis, necroptosis, ferroptosis, autophagic cell death, cellular energy catastrophe and others.

In conclusion, the setting of our I/R experiment is unique because it is performed inside the human body. It mimics conditions where the uterus would be removed from a deceased donor after experiencing long warm ischemia because of its delayed retrieval after removal of life-saving organs. Long warm *in situ* I/R appears to be well tolerated by the human uterus. However, it triggers significant cell death that becomes apparent during storage. Inclusion of Gap27 during cold storage could be useful to protect the uterus obtained from deceased donors where marked warm ischemia is likely because priority retrieval of life-saving organs. This study is a promising lead to the discovery of new measures for uterus protection during the long period between organ retrieval and re-implantation, which may promote implantation success.

## References

[1] Brännström M, Diaz-Garcia C, Hanafy A, Olausson M, Tzakis A. Uterus transplantation: animal research and human possibilities. Fertil Steril. 2012 6 1;97(6):1269–76. 10.1016/j.fertnstert.2012.04.001 22542990

[2] Brännström M. Uterus transplantation and beyond. J Mater Sci Mater Med. 2017 5; 28(5):70 10.1007/s10856-017-5872-0 28357688 PMC5371630

[3] Brännström M, Johannesson L, Bokström H, Kvarnström N, Mölne J, Dahm-Kähler P, et al Livebirth after uterus transplantation. Lancet. 2015;385(9968):607–16. 10.1016/S0140-6736(14)61728-1 25301505

[4] Puntambekar S, Telang M, Kulkarni P, Jadhav S, Sathe R, Warty N, et al Laparoscopic-Assisted Uterus Retrieval From Live Organ Donors for Uterine Transplant. J Minim Invasive Gynecol. 2018;25(4):571–2. 10.1016/j.jmig.2017.11.001 29133152

[5] Testa G, McKenna GJ, Gunby RT, Anthony T, Koon EC, Warren AM, et al First live birth after uterus transplantation in the United States. Am J Transplant. 2018 5 1;18(5):1270–4. 10.1111/ajt.14737 29575738

[6] Flyckt R, Falcone T, Quintini C, Perni U, Eghtesad B, Richards EG, et al First birth from a deceased donor uterus in the United States: from severe graft rejection to successful cesarean delivery. Am J Obstet Gynecol. 2020 8 1;223(2):143–51. 10.1016/j.ajog.2020.03.001 32151611

[7] Ozkan O, Akar ME, Ozkan O, Erdogan O, Hadimioglu N, Yilmaz M, et al Preliminary results of the first human uterus transplantation from a multiorgan donor. Fertil Steril. 2013 2 1;99(2):470–476.e5. 10.1016/j.fertnstert.2012.09.035 23084266

[8] Ejzenberg D, Andraus W, Baratelli Carelli Mendes LR, Ducatti L, Song A, Tanigawa R, et al Livebirth after uterus transplantation from a deceased donor in a recipient with uterine infertility. Lancet. 2018 12 4. 10.1016/S0140-6736(18)31766-5 30527853

[9] Wranning CA, Mölne J, El-akouri RR, Kurlberg G, Brännström M. Short-term ischaemic storage of human uterine myometrium—basic studies towards uterine transplantation. Hum Reprod. 2005;20(10):2736–44. 10.1093/humrep/dei125 15980004

[10] de Groot H, Rauen U. Ischemia-reperfusion injury: processes in pathogenetic networks: a review. Transplant Proc. 2007 3 1;39(2):481–4. 10.1016/j.transproceed.2006.12.012 17362763

[11] Thompson RJ, Zhou N, MacVicar B a. Ischemia opens neuronal gap junction hemichannels. Science. 2006 5 12;312(5775):924–7. 10.1126/science.1126241 16690868

[12] García-Dorado D, Rodríguez-Sinovas A, Ruiz-Meana M. Gap junction-mediated spread of cell injury and death during myocardial ischemia–reperfusion. Cardiovasc Res. 2004 2 15;61(3):386–401. 10.1016/j.cardiores.2003.11.039 14962471

[13] Gadicherla AK, Wang N, Bulic M, Agullo-Pascual E, Lissoni A, De Smet M, et al Mitochondrial Cx43 hemichannels contribute to mitochondrial calcium entry and cell death in the heart. Basic Res Cardiol. 2017 5 31;112(3):27 10.1007/s00395-017-0618-1 28364353

[14] Cea LA, Puebla C, Cisterna BA, Escamilla R, Vargas AA, Frank M, et al Fast skeletal myofibers of mdx mouse, model of Duchenne muscular dystrophy, express connexin hemichannels that lead to apoptosis. Cell Mol Life Sci. 2016 7 23;73(13):2583–99. 10.1007/s00018-016-2132-2 26803842 PMC11108387

[15] Winterhager E, Kidder GM. Gap junction connexins in female reproductive organs: implications for women’s reproductive health. Hum Reprod Update. 2015;0(0):1–13. 10.1093/humupd/dmv007 25667189

[16] Alexander DB, Goldberg GS. Transfer of biologically important molecules between cells through gap junction channels. Curr Med Chem. 2003 10 1;10(19):2045–58. 10.2174/0929867033456927 12871102

[17] Saez JC, Berthoud VM, Branes MC, Martinez AD, Beyer EC. Plasma Membrane Channels Formed by Connexins: Their Regulation and Functions. Physiol Rev. 2003 10; 83(4):1359–400. 10.1152/physrev.00007.2003 14506308

[18] Decrock E, Vinken M, De Vuyst E, Krysko DV, D ‘herde K, Vanhaecke T, et al Connexin-related signaling in cell death: to live or let die? Cell Death Differ. 2009 4; 16(4):524–36. 10.1038/cdd.2008.196 19197295

[19] Wang N, De Bock M, Decrock E, Bol M, Gadicherla A, Vinken M, et al Paracrine signaling through plasma membrane hemichannels. Vol. 1828, Biochim. Biophys. Acta 2013; 1828(1): 35–50. 10.1016/j.bbamem.2012.07.002 22796188 PMC3666170

[20] Retamal MA, Sáez JC. Hemichannels; from the molecule to the function. Front Physiol. 2014;5:411 10.3389/fphys.2014.00411 25368583 PMC4202783

[21] Wang N, De Vuyst E, Ponsaerts R, Boengler K, Palacios-Prado N, Wauman J, et al Selective inhibition of Cx43 hemichannels by Gap19 and its impact on myocardial ischemia/reperfusion injury. Basic Res Cardiol. 2013 1;108(1):309 10.1007/s00395-012-0309-x 23184389 PMC3666173

[22] Hawat G, Benderdour M, Rousseau G, Baroudi G. Connexin 43 mimetic peptide Gap26 confers protection to intact heart against myocardial ischemia injury. Pflügers Arch—Eur J Physiol. 2010 8 1;460(3):583–92. 10.1007/s00424-010-0849-6 20514543

[23] Bol M, Van Geyt C, Baert S, Decrock E, Wang N, De Bock M, et al Inhibiting connexin channels protects against cryopreservation-induced cell death in human blood vessels. Eur J Vasc Endovasc Surg. 2013 4;45(4):382–90. 10.1016/j.ejvs.2012.12.012 23352273

[24] Wranning Caiza A., Dahm-Kähler P, Mölne J, NILSSON UA, ENSKOG A, et al Transplantation of the uterus in the sheep: oxidative stress and reperfusion injury after short-time cold storage. Fertil Steril. 2008 9 1;90(3):817–26. 10.1016/j.fertnstert.2007.07.1340 17904131

[25] Díaz-García C, Johannesson L, Enskog a, Tzakis a, Olausson M, Brännström M. Uterine transplantation research: laboratory protocols for clinical application. Mol Hum Reprod. 2012 2; 18(2):68–78. 10.1093/molehr/gar055 21900333

[26] Chaytor AT, Evans WH, Griffith TM. Peptides homologous to extracellular loop motifs of connexin 43 reversibly abolish rhythmic contractile activity in rabbit arteries. J Physiol. 1997 8 15;503(1):99–110. 10.1111/j.1469-7793.1997.099bi.x 9288678 PMC1159890

[27] Tang HM, Tang HL. Anastasis: Recovery from the brink of cell death. Vol. 5, R Soc Open Sci. 2018;5:180442 10.1098/rsos.180442 30839720 PMC6170572

[28] Tang HL, Tang HM, Mak KH, Hu S, Wang SS, Wong KM, et al Cell survival, DNA damage, and oncogenic transformation after a transient and reversible apoptotic response. Mol Biol Cell. 2012 6 15;23(12):2240–52. 10.1091/mbc.E11-11-0926 22535522 PMC3374744

[29] Ichim G, Lopez J, Ahmed SU, Muthalagu N, Giampazolias E, Delgado ME, et al Limited Mitochondrial Permeabilization Causes DNA Damage and Genomic Instability in the Absence of Cell Death. Mol Cell. 2015 3 5;57(5):860–72. 10.1016/j.molcel.2015.01.018 25702873 PMC4352766

[30] Laskowski I, Pratschke J, Wilhelm MJ, Gasser M, Tilney NL. Molecular and cellular events associated with ischemia/reperfusion injury. Ann Transplant. 2000;5(4):29–35. 11499357

[31] Hauet T, Goujon JM, Vandewalle A. To what extent can limiting cold ischaemia/reperfusion injury prevent delayed graft function? Nephrol Dial Transplant. 2001 10 1;16(10):1982–5. 10.1093/ndt/16.10.1982 11572883

[32] Zitkute V, Kvietkauskas M, Leber B, Strupas K, Stiegler P, Schemmer P. Ischemia and reperfusion injury in uterus transplantation: A comprehensive review. Vol. 34, Transplant Rev. 2020;34(3):100550 10.1016/j.trre.2020.100550 32498979

[33] Adachi M, Kisu I, Nagai T, Emoto K, Banno K, Umene K, et al Evaluation of allowable time and histopathological changes in warm ischemia of the uterus in cynomolgus monkey as a model for uterus transplantation. Acta Obstet Gynecol Scand. 2016 9 1;95(9):991–8. 10.1111/aogs.12943 27329637

[34] Kisu I, Umene K, Adachi M, Emoto K, Nogami Y, Banno K, et al Allowable warm ischemic time and morphological and biochemical changes in uterine ischemia/ reperfusion injury in cynomolgus macaque: a basic study for uterus transplantation. Hum Reprod Adv Access Publ August. 2017;32(31):2026–35. 10.1093/humrep/dex250 28938750

[35] Brännström M, Johannesson L, Dahm-Kähler P, Enskog A, Mölne J, Kvarnström N, et al First clinical uterus transplantation trial: A six-month report. Fertil Steril. 2014;101(5):1228–36. 10.1016/j.fertnstert.2014.02.024 24582522

[36] Brännström M, Dahm-Kähler P, Ekberg J, Akouri R, Groth K, Enskog A, et al Outcome of Recipient Surgery and 6-Month Follow-Up of the Swedish Live Donor Robotic Uterus Transplantation Trial. J Clin Med. 2020 7 22;9(8):2338 10.3390/jcm9082338 32707899 PMC7464615

[37] Kwon Y-S, Jung D-Y, Lee S-H, Ahn JW, Roh HJ, Im KS. Transient Occlusion of Uterine Arteries with Endoscopic Vascular Clip Preceding Laparoscopic Myomectomy. J Laparoendosc Adv Surg Tech. 2013 8 31;23(8):679–83. 10.1089/lap.2012.0540 23631666

[38] Liu L, Li Y, Xu H, Chen Y, Zhang G, Liang Z. Laparoscopic transient uterine artery occlusion and myomectomy for symptomatic uterine myoma. Fertil Steril. 2011 1 1;95(1):254–8. 10.1016/j.fertnstert.2010.05.006 21168582

[39] Wang CJ, Yuen LT, Han CM, Kay N, Lee CL, Soong YK. A transient blocking uterine perfusion procedure to decrease operative blood loss in laparoscopic myomectomy. Chang Gung Med J. 2008;31(5):463–8. 19097593

[40] Del Priore G, Stega J, Sieunarine K, Ungar L, Smith JR. Human uterus retrieval from a multi-organ donor. Obstet Gynecol. 2007 1 1;109(1):101–4. 10.1097/01.AOG.0000248535.58004.2f 17197594

[41] Sieunarine K, Lindsay I, Ungar L, Del Priore G, Smith JR. Cold ischaemic preservation of human uterine tissue. Int Surg. 2009;93(6):366–72. 20085047

[42] Finnegan R, Zhu P, Stephenson S, Nadig S, Atkinson C. Cold Storage Stabilization of Gap Junctions Reduces Post Transplant Ischemia Reperfusion Injury. J Hear Lung Transplant. 2016 4 1;35(4):S160–1.

[43] Goncharenko K, Eftekharpour E, Velumian AA, Carlen PL, Fehlings MG. Changes in gap junction expression and function following ischemic injury of spinal cord white matter. J Neurophysiol. 2014 11 1;112(9):2067–75. 10.1152/jn.00037.2013 25080569 PMC4274924

[44] Feine I, Pinkas I, Salomon Y, Scherz A. Local oxidative stress expansion through endothelial cells—a key role for gap junction intercellular communication. PLoS One. 2012;7(7):e41633 10.1371/journal.pone.0041633 22911831 PMC3402439

[45] Contreras JE, Sánchez HA, Véliz LP, Bukauskas FF, Bennett MVL, Sáez JC. Role of connexin-based gap junction channels and hemichannels in ischemia-induced cell death in nervous tissue. Brain Res Rev. 2004 12 1;47(1–3):290–303. 10.1016/j.brainresrev.2004.08.002 15572178 PMC3651737

[46] Hawat G, Hélie P, Baroudi G. Single intravenous low-dose injections of connexin 43 mimetic peptides protect ischemic heart in vivo against myocardial infarction. J Mol Cell Cardiol. 2012 10; 53(4):559–66. 10.1016/j.yjmcc.2012.07.008 22841862

[47] Li X, Zhao H, Tan X, Kostrzewa RM, Du G, Chen Y, et al Inhibition of connexin43 improves functional recovery after ischemic brain injury in neonatal rats. Glia. 2015 9 1;63(9):1553–67. 10.1002/glia.22826 25988944

